# The nexus between circular economy innovation, market competitiveness, and triple bottom lines efficiencies among SMEs: evidence from emerging economies

**DOI:** 10.1007/s11356-023-30956-0

**Published:** 2023-11-15

**Authors:** Fazal Ur Rehman, Solomon Gyamfi, Samma Faiz Rasool, Fazal Akbar, Khawar Hussain, Viktor Prokop

**Affiliations:** 1 Science and Research Centre, Faculty of Economics and Administration, University of Pardubice, 53210 Pardubice, Czech Republic; 2 College of Business Administration, Al Yamamah University, Riyadh, Kingdom of Saudi Arabia; 3 Faculty of Technology Management and Business, Universiti Tun Hussein Onn Malaysia, Batu Pahat, Malaysia

**Keywords:** Circular economy innovation, Market competitiveness, TBL efficiencies, SMEs

## Abstract

Recently, the trend of circular economy innovation (CEI) drive SMEs to initiate more sustainable practices to attain market competitiveness but rare attention has been paid in emerging economies. Hence, this study plans to explore the impacts of CEI on the triple bottom lines (TBL) efficiencies in the light of market competitiveness among the production SMEs in emerging economies. The study complied data by using a survey-based technique in Pakistan, Malaysia, and China. With a sample of 306 for each segment, data were evaluated with PLS-SEM to clarify results. The findings reveal that CEI has positive significant effects on the market competitiveness and TBL efficiencies among the production SMEs in emerging economies. The findings also clarify that market competitiveness mediates the relationship between CEI and the TBL efficiencies. The findings elaborate the theoretical foundations for environmental-based production SMEs to formulate more sustainable strategies in the light of CEI to gain market competitiveness. It also clarifies the understandings of policy makers and environmental regulators by providing a novel precursor to frame the environment-based TBL guidelines for SMEs. It adds to the UN sustainability agenda by elevating the role of CEI as a novel domain among emerging economies grounded on the resource-based view theory.

## Introduction

The UN sustainability objectives have grasped significant interest to accentuate the reputation of scare natural resources, avoid environmental deprivation, and reduce poverty and hunger in the world (UN 2015). This core idea has introduced the perception of TBL efficiencies in industrial world and perceived as a main driver of market competitiveness (Mwangi et al. 2021). Because of its significance and sustainability measures, numerous international firms have initiated the practices of sustainable development and circular economy (Lii and Kuo 2016; García-Sánchez et al. 2021; Prokop et al. 2022). Circular economy is the closed-loop, ecodesign, or economic system that intends to maximize the usage and life of resources and materials and minimize the wastage of these scare natural resources (Deutz 2020). CEI is the transition from linear economic models based on take, make, use, and waste toward circular models that minimize, recover, recycle, and reuse materials, water, and energy to meet the sustainable economic, environmental, and social needs in an efficient way among societies on continuous basis (Rehman et al. 2022). Instead of its greater importance, SMEs have shown lower engagement in the initiatives of sustainability and CEI due to limited sources, lack of knowledge, and rare interest in the green practices (Sullivan-Taylor and Branicki 2011; Johnson and Schaltegger 2016; Lopez-Perez et al. 2017; Korsakienė and Raišienė 2022; Rehman et al. 2022), and even have a bigger contribution in the world economy (Garetti and Taisch 2012; Asgary et al. 2020; Rodríguez-Espíndola et al. 2022a), and wide impacts on the surrounding atmosphere (Miller et al. 2011; OECD 2018a, 2018b; Rodríguez-Espíndola et al. 2022b). Therefore, it is essential to uncover the factors of lower engagement, characteristics, competencies, and context of SMEs, and encourage them toward sustainability and circularity practices (Parker et al. 2009; Klewitz and Hansen 2014; Syromyatnikov et al. 2021; Prokop et al. 2023), specially, the TBL efficiencies in emerging economies.

Broadly, the firms are adopting the innovative routes to travel from the outmoded means of business toward sustainable production to ensure the energy preservation, save the scare natural resources, protect the green belt in societies, and achieve the TBL efficiencies. The triple bottom line (TBL) concept is the integration of economics, environmental, and social dimensions of sustainability orientation in emerging literature (Sarkis and Dhavale 2015; Svensson et al. 2018), and the combination of these three factors usually called as TBL approach. However, due to the UN sustainability objectives, firms are eagerly searching for the initiatives of circular economy to keep balance in the TBL efficiencies and sustainability-oriented business practices to achieve the long-lasting business objectives and market competitiveness. Market competitiveness “is the extent of the competitiveness of the operating environment in which a service firm operates” (Yee et al. 2013), and is the ability of firm to compete in a particular market to attain higher relative position. For the reason, the green initiatives and CEI are perceived as the key sources to achieve TBL efficiencies, growth, create values, and improve market competitiveness (Dey et al. 2020; Bucea-Manea-Țoniş et al. 2021; Barros et al. 2021; Rodríguez-Espíndola et al. 2022a; Peterková et al. 2022; Karman et al. 2023), as the practices of CEI have altered the business environment from traditional setup to more sustainable business practices to attain the TBL efficiencies, enhance revenue-making paradigm, and market competitiveness (Barros et al. 2021; Rehman et al. 2022). The practices of CEI are applied as a solution-based strategy to reduce losses of resources and achieve TBL efficiencies and market competitiveness, and is a key driver of sustainability (Abad-Segura et al. 2020; Ntsonde and Aggeri 2021). Concisely, the trend of CEI has emerged the closed-loop business activities and convert the business setup to sustainable business activities (Brown et al. 2019) that ultimately lead to obtain TBL efficiencies and market competitiveness (Rodríguez-Espíndola et al. 2022a). These environmental-based strategies are implemented to ensure the sustainable production, cleaner production, healthy drinking water, and living style among societies; gain market competitiveness; and advance eco-system (Cracolici et al. 2018; Sehnem et al. 2019; Abad-Segura et al. 2020).

But instead of substantial efforts, the trend of CEI is novel, having rare literature, and need further investigation to gain TBL efficiencies, market competitiveness, and direct the policy makers and practitioners (Ghisellini et al. 2016; Ormazabal et al. 2018; Rodríguez-Espíndola et al. 2022b). Particularly, the prior research has paid limited attention to explore the role of CEI in the various business areas and different organizational context (Barros et al. 2021), and needs further investigation to design a more sustainable business model to attain market competitiveness and TBL efficiencies, especially there are calls for further research in these domains among SMEs (Salvador et al. 2021; Rodríguez-Espíndola et al. 2022a; Ting et al. 2023). Precisely, the importance of circularity, transition process, CEI strategies’ role in TBL efficiencies, and market competitiveness is not so clear and required deep investigation (Awan et al. 2021; Groening et al. 2018; Hudecheck et al. 2020; Ranjbari et al. 2021; Wang et al. 2018; White et al. 2019; Cronin et al. 2011; Dangelico and Vocalelli 2017; Moraga et al. 2021; Papadas et al. 2019), especially in the production SMEs in emerging market like Pakistan, Malaysia, and China. Specifically, the mediating role of market competitiveness between the CEI and the TBL efficiencies among the production SMEs in emerging economies has not been explored so far.

Concisely, the management of resources is a main concern of success among production SMEs (Rasool et al. 2023), and a considerable challenge in transition process. Moreover, there are worst climate change and environmental issues in Pakistan (Mir et al. 2022), but still there is lack of interest to adopt the circular economy initiatives among firms in Pakistan (Uddin et al. 2023). Similarly, the issues of warming and rainfall irregularities have created serious climate problems in Malaysian society (Tang 2019), but the initiatives of circular economy are widely launched in public sector and private sector face bundle of barriers (Ting et al. 2023), and widely ignored in Malaysia. Further, China is seen as a largest polluter in terms of greenhouse gas emissions among emerging economies and needs extensive research in the policy drivers of circular economy to explore the success and failure factors (Bleischwitz et al. 2022). Hence, the current study has discovered a gap of knowledge to investigate the nexus between CEI, market competitiveness, and TBL efficiencies among the production SMEs in emerging economies. Thus, we aim to address the following shortcomings:

1. What is the impact of CEI on the TBL efficiencies among the production SMEs in emerging economies?
2. What is the influence of CEI on market competitiveness among the production SMEs in emerging economies?
3. Does market competitiveness drive TBL efficiencies among the production SMEs in emerging economies?
4. Does market competitiveness mediate between CEI and TBL efficiencies among the production SMEs in emerging economies?

Subsequently, to establish alliance among defined variables to fill up the risen research gap, we required to conduct a study in the emerging economies. Hence, the integration of CEI, market competitiveness, and TBL efficiencies among the production SMEs backed up the prior research and provides a theoretical foundation for the environment-based competitive strategies. The novel precursor provides a route of TBL efficiencies for the production SMEs to define more interesting strategies for market competitiveness in the light of CEI. It also complements by examining the mediating role of market competitiveness between CEI and TBL efficiencies in the production SMEs. It also extends the prior research and contributes by integrating the literature of CEI, market competitiveness, and TBL efficiencies among the production SMEs grounded on the resource-based view theory in emerging economies. Further, the findings show that the practices of CEI trigger all the three dimensions of TBL approach among production SMEs and illustrate the pathways that drive the sustainability initiative to create values for societies. The study explored some differences in TBL efficiencies among emerging economies, so the managers can realize the magnitudes of influential factors to formulate more effective business and environmental strategies. Finally, the practitioners and policy makers can exploit our findings to wisely utilize their available and scare resources and mitigate integrative strategies to reduce the loss of these scare resources and add in the UN sustainability agenda. However, the structure of the study starts with introduction followed by literature review and hypothesis development. Methodological procedure is explained in the third while results in the fourth slice. Discussion of results, implications for policy, and summary of findings is placed at the end of study.

## Theoretical framework and hypothesis development

### Resource-based view theory

The resource-based view theory was defined by Barney 1991 and is a widely used managerial framework in emerging literature. The theory focuses to exploit the strategic internal resources, capabilities, and core competencies among firms to achieve a sustainable market competitiveness. It assesses the strategic fit of capabilities and key resources that are value, rarity, imperfect imitability, and lack of substitutability to attain desirable market competitiveness (Kshetri 2008). It helps to exploit the key opportunities and detect the environmental threats to improve efficiencies and market competitiveness. Hence, the analysis of the related literature focuses on the elucidation of the ways where SMEs through CEI perform on the economic, social, and environmental fronts powered by their market competitiveness.

### CEI, TBL efficiencies, and the resource-based view theory

Sustainable development has become a policy yardstick in the quest to enhancing the development of modern economies. In effect, multifaceted interests from the international organizations, agencies, and countries are seeking to bolster sustainability resulting in the creation of ambitious agendas and goals both from the macro and micro perspectives of the economies. The key aim has been to revise the TBL practices to ensure peace and prosperity around the world (Rodríguez-Antón et al. 2022). This can be achieved through the elimination of waste and inefficiencies of the linear economy by speed up the journey toward circular economy (Rodríguez-Antón and Alonso-Almeida 2018; Rodríguez-Antón 2019). Circular economy (CE) designates the economic system that is rooted on business models, which concerns the reduction of economic resources loss, alternative reusing, recycling, and recovering of material resources in production/distribution and consumption processes of the economic system (Kirchherr et al. 2017).

CE approach is considered an economic route to reach TBL efficiencies, and it relies on the novel business strategies and market interactions (Valverde and Avilés-Palacios 2021). As a mean to integrate circular economy practices in manufacturing systems, Ly (2021) contend is built on circular use of resources to achieve TBL efficiencies among SMEs. Valverde and Avilés-Palacios (2021) opine that it is particularly useful for achieving TBL efficiencies. Circular economy is a key practice to realize the TBL efficiencies and ecological well-being (Valverde and Avilés-Palacios 2021). Consequently, the application of CEI has significant influence on SDGs, including TBL efficiencies, as well as international competitiveness (DeCajas and Seguros 2013). CEI is a strategic decision to meet TBL efficiencies while achieving sustainability goals (Lewandowski 2016). CE and the perception of resource-based view (RBV) instituted by Barney 1991 are interconnected in promoting sustainable economic growth and addressing environmental challenges.

Nevertheless, in the wisdom of the tenets of the RBV, SMEs can adopt CEI, which ensures reduction in costs, improving resource efficiency, and enhancing the exploration of their organizational resources (Joyce and Winch 2004; Horbach et al. 2022) for market competitiveness. However, to achieve a sustainable market competitiveness, organizations (SMEs) can recognize the unique qualities of their available resources, specifically their value, rarity, imperfectability, and non-substitutability, and utilize them in such a manner that enhances organizational efficiency and effectiveness. Such resources can help SMEs to gain market competitiveness and create barriers for competitors. Consequently, by adopting CEI practices, firms can leverage the resource-based view theory to achieve market competitiveness, as well as TBL efficiencies. This is because CEI is considered the key route toward sustainable imminent, and to advance the economic system (Khajuria 2020; Valverde and Avilés-Palacios 2021), but needs further investigation. Specifically, the initiatives of circular economy have positive effects on the TBL efficiencies (Rodríguez-Espíndola et al. 2022a; Rehman et al. 2022; Wiebe et al. 2023), but more investigation is needed to develop a solid theoretical framework for CEI in the context of TBL efficiencies (Suárez-Eiroa et al. 2019; Rodríguez-Antón et al. 2022), which are considered the top priorities among SDGs according to the UN 2030 sustainability agenda. Therefore, we can hypothesize that:

**H1:**CEI triggers the TBL efficiencies among the production SMEs in emerging economies.

### CEI and market competitiveness

SMEs are essential to the economy since they account for a large portion of employment and economic growth. However, SMEs confront several difficulties, such as lack of innovation skills, restricted access to financing, and resource limitations. As a result, SMEs must use novel strategies to acquire market competitiveness and realize sustainability objectives. Usually, innovation activities enhance firm performance and market competitiveness (Mulkay 2019). So by giving SMEs the chance to cut costs, increase resource efficiency, and strengthen their brand name, CEI offers a viable answer to these problems. Prior studies suggest that SMEs are increasingly adopting CEI strategies to help them reach this goal. The goal is to produce, deliver, and collect value for societies specially in the TBL context and market competitiveness (Suchek et al. 2021; Barros et al. 2021). In addition to enhancing societal well-being, SMEs are concentrating to gain market competitiveness through creative solutions and close collaboration in the development of the circular economy, which boosts productivity and adds value (Razminiene and Tvaronaviciene 2018; Köhler et al. 2022). In order to gain a long-lasting market competitiveness, businesses are frequently pushed to implement the strategies of CEI (Jakhar et al. 2019; Herrero-Luna et al. 2022). As the adoption of CEI by SMEs can increase their capacity to achieve sustainable development and desired levels of market competitiveness, in accordance with existing research, which suggests that circular economy practices like reverse, reuse, reworking, and recycling can enhance market competitiveness (Spring and Araujo 2017; Giannetti et al. 2020; Soh and Wong 2021). SMEs usually adopt CEI practices to ensure their growth and gain market competitiveness (Ly 2021), provide an edge, and help them to achieve their strategic objectives (Hart and Milstein 2003). According to the previous literature, adopting CEI strategies can boost firms’ profit margins, lead to gain market competitiveness, motivate them to produce and sell more than their competitors, and promote internalization and externalization (Ly 2021) while maintaining the long-term survival of firms and meeting the sustainable demands of the society (Prieto-Sandoval et al. 2019), as the initiatives of CEI and environmental practices can stimulate the innovation activities among firms and improve its market competitiveness (Porter 1996; Rehman et al. 2022). Particularly, the transition toward CEI can improve the firm’s growth, bring technical change, and enhance the firms’ technical capabilities and competencies (Kinugasa 1998). Barros et al. (2021) observed that the circular economy initiatives have positive influence on the market competitiveness. In the same way, Rodríguez-Espíndola et al. (2022a) noted that circular economy initiatives have positive influence on the market competitiveness. Although various studies have examined the association between CEI and market competitiveness, prior studies have paid limited attention to explore the relationship between CEI and market competitiveness in a comparative context among the production SMEs in emerging economies. Hence, we hypothesize that:

**H2:**CEI triggers market competitiveness among the production SMEs in emerging economies.

### Market competitiveness and TBL efficiencies

The pursuit of market competitiveness has heightened the corporate rivalry in the age of globalization and attracted considerable attention from practitioners and relevant literature. Market competitiveness is viewed as a source of assurance, profit margin, market shares, and investors’ focus, establishing a brand image in targeted communities (Batista et al. 2016; Hurnyak et al. 2022). Innovative tactics, distinctive products, and services can draw people in, create market monopolies, and provide businesses an advantage over competitors, all of which eventually lead to TBL efficiencies (Anwar 2018). In order to improve long-term efficiencies and gain market competitiveness, businesses implement a variety of strategies (such as cost leadership, product and service innovation, CEI) that ultimately improves the TBL efficiencies among SMEs. A higher market competitiveness can result in higher TBL efficiencies, and vice versa, which can indirectly result in winning situation (Anwar 2018). As a result, businesses with innovative tactics and distinctive capabilities can increase their profitability, build their brand, and have a stronger market competitiveness, all of which attest to their superior TBL efficiencies. Schulz and Flanigan (2016) found that market competitiveness improves the TBL efficiencies among firms. Ye et al. (2015) also observed that market competitiveness affects the TBL efficiencies in the construction sector. Like this, earlier research (Lechner and Gudmundsson 2014; Bapat and Mazumdar 2015; Saeidi et al. 2015) have identified considerable effects of market competitiveness on the firm’s performance; however, it failed to concentrate on the analysis to investigate the role of market competitiveness in the TBL efficiencies among the production SMEs, especially in a comparative context among emerging economies. Therefore, in this study, we hypothesize that:

**H3:**Market competitiveness trigger the TBL efficiencies among production SMEs in the emerging economies.

### Mediating role of market competitiveness between CEI and TBL efficiencies

Businesses are heavily emphasizing on creating values for societies and numerous stakeholders in the sustainable environment to boost their profit margins and assure their existence. In this scenario, businesses are using a variety of techniques to provide customers value and gain market competitiveness to secure profit margin (Teece 2010; Anwar 2018). For instance, CEI is seen as a key factor in achieving sustainable development goals and enhances TBL efficiencies to produce values for societies (Valverde and Avilés-Palacios 2021). Since companies’ skills lead to improve performance, particularly, SMEs need to develop effective strategies to achieve market competitiveness and demonstrate superior TBL efficiencies in the view of intense competition (Pucci et al. 2017; Anwar 2018). However, Anwar et al. (2018) has observed that competitive advantage mediates between the business model innovation and SMEs performance.

Similarly, Rua et al. (2018) investigated the mediating role of market competitiveness between the primary factors of SMEs export operations (entrepreneurial orientation, intangible resource, and absorptive capacities). Additionally, Udriyah et al. (2019) investigated the mediating role of market competitiveness between market orientation and innovation with SME performance. Udriyah et al. (2019) found the performance of SMEs to be significantly impacted by market orientation as well as innovation indirectly through market competitiveness. Likewise, Yasa et al. (2020) investigated the mediating role of market competitiveness between promotional strategy and the marketing performance among SMEs. Rehman (2023) examined the mediating role of market competitiveness between the management practices and firm innovation. Nevertheless, earlier research has never examined the mediating role of market competitiveness between CEI and TBL efficiencies among the production SMEs especially in a comparative context among emerging economies. Therefore, we hypothesize that:

**H4:**Market competitiveness mediates between CEI and the TBL efficiencies among the production SMEs in emerging economies.

**Fig. 1:**
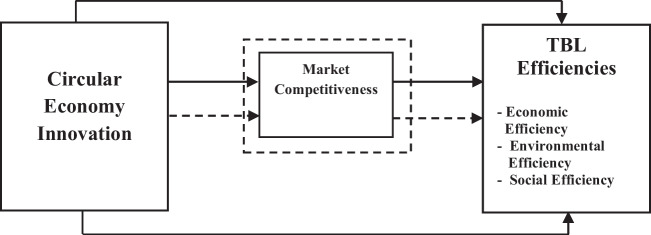
Theoretical framework

Based on the review of above literature, this study lays the foundation of conceptual model including CEI, market competitiveness, and TBL efficiencies as display in (Fig. 1).

## Methodology

A questionnaire-based survey was applied to collect data among the production SMEs in Pakistan, Malaysia, and China. The questionnaire-based survey approach is widely used and perceived useful and easy to use, helpful to avoid misunderstandings, display statistical outcomes, and more reliable to get accurate data (Chaudhry et al. 2023; Rind et al. 2022). However, these countries were selected due to the environmental issues and lack of practices in circular economy initiatives among SMEs, due to the need to outline the solid policy drivers to fixate the success and failure factors (Bleischwitz et al. 2022; Uddin et al. 2023; Ting et al. 2023), and due to the limited resources for data collection. Participants were the owners, CEO, and managerial staff members of the registered production SMEs among emerging economies. The sample specification was determined using G-Power that is a valid approach for PLS-SEM studies (Hair et al. 2016). The outcomes indicate that 119 is the minimum number to draw results of each data set. However, 306 questionnaires were randomly circulated in each segment but only 270 responses were considered due to the accuracy in each data set. It was also ensured that the data is only gathered for research purposes.

The scale was adopted and adapted based on the prior studies (Table 1) and was close ended based on the Likert scale from strongly agree to strongly disagree. Later, the expert researchers checked the accuracy of scale and confirmed its validity. At the same time, a pilot study was conducted to evaluate the reliability of the instrument (Kain 2007; ur Rehman et al. 2018) as a pilot study is usually conducted to test the reliability of scale. Some items were deleted due to its weakness and a more valid scale was operationalized after pilot study. The scale was developed in the English language and the research team helped to clarify the respondents’ understanding about questionnaires and research in case of any confusions. For the final results, data was screened and cleaned to check the missing values, outliers, normality, multicollinearity, etc. and the problematic responses were deleted accordingly. After that, Smart Partial Least Square Structural Equation Modeling (PLS-SEM) was applied to confirm the hypothesis that has the advantage to validate each step in systematic way and display results only in one click, and has better performance in predictive studies (Rehman et al. 2021; Kabra et al. 2017; Rehman 2023; Rasool et al. 2023). PLS-SEM has no hard issues of data normality, handle complex model, and preferable in formative models (Rehman 2019), and was considered suitable in this study.

**Table 1:** Scale of measurement

Constructs		Number of items	Authors
CEI		8	Rodríguez-Espíndola et al. (2022b); Rehman et al. (2023a)
Market competitiveness		8	Anwar et al. (2018)
TBL efficiencies	Economics efficiency	8	Rizwanullah et al. (2021); Anwar et al. (2020)
	Environmental efficiency	12	Rizwanullah et al. (2021); Anwar et al. (2020)
	Social efficiency	7	Rehman and Anwar (2019); Anwar et al. (2018)

## Results

The study applied PLS-SEM to assess the collected data to draw the required outcomes. Usually in PLS-SEM, the procedure of measurement model is applied to examine the factors loading, composite reliability, AVE, discriminant validity, and HTMT or cross loading to validate the measure accuracy in the model (Zeb et al. 2020; Rehman 2023), while the technique of structural model is applied to confirm the formulated hypothesis (Ringle et al. 2015; Rehman and Zeb 2023a).

As per the rules of PLS-SEM authentication, the values of factor loading should be 0.7 or higher, composite reliability 0.7 or greater, and AVE 0.5 or greater (Zeb et al. 2021; Rehman and Zeb 2023b) as indicated in Table 2. The findings show that the concerned outcomes exist in the acceptable range. The findings have authenticated the recommended procedure of measurement model and structural model, and verified the validation of theoretical framework. Moreover, the methodological procedure of Fornell and Larcker (1981) was followed to verify the precision of discriminant validity as shown in (Table 3). The results indicate that the slanted values are better as compared to other factors and further verified with the test of HTMT as indicated in (Table 3), and with the technique of cross loadings as presented in Appendix 1.

**Table 2:** Measurement model

Variable	Items	Pakistan					Malaysia					China				
		F.L	CA	rho_A	C.R	AVE	F.L	CA	rho_A	C.R	AVE	F.L	CA	rho_A	C.R	AVE
Circular economy innovation (CEI)	CEI1	0.827	0.909	0.916	0.926	0.612	0.692	0.902	0.909	0.922	0.599	0.703	0.900	0.906	0.92	0.59
	CEI 2	0.790					0.656					0.782				
	CEI 3	0.730					0.886					0.709				
	CEI4	0.758					0.772					0.790				
	CEI 5	0.683					0.885					0.890				
	CEI6	0.773					0.758					0.729				
	CEI 7	0.893					0.737					0.780				
	CEI8	0.787					0.776					0.745				
Market competitiveness (MC)	MC1	0.818	0.904	0.912	0.922	0.597	0.793	0.891	0.912	0.910	0.559	0.817	0.910	0.914	0.927	0.614
	MC2	0.729					0.681					0.758				
	MC3	0.851					0.804					0.853				
	MC4	0.749					0.685					0.772				
	MC5	0.778					0.731					0.806				
	MC6	0.771					0.720					0.817				
	MC7	0.761					0.811					0.771				
	MC8	0.715					0.746					0.662				
Economics performance (EP)	EP1	0.539	0.855	0.878	0.889	0.537	0.724	0.911	0.922	0.931	0.661	0.812	0.904	0.905	0.924	0.636
	EP2	0.721					0.938					0.841				
	EP3	0.684					0.732					0.823				
	EP4	0.805					0.729					0.736				
	EP5	0.833					0.920					0.797				
	EP6	0.741					0.691					0.821				
	EP7	0.769					0.914					0.744				
Environmental performance (ENP)	ENP1	0.948	0.927	0.931	0.943	0.707	0.785	0.894	0.907	0.917	0.613	0.815	0.903	0.904	0.921	0.634
	ENP2	0.733					0.710					0.817				
	ENP3	0.696					0.803					0.718				
	ENP4	0.927					0.894					0.787				
	ENP5	0.688					0.733					0.792				
	ENP6	0.936					0.791					0.816				
	ENP7	0.904					0.751					0.825				
Social performance (SP)	SP1	0.655	0.896	0.898	0.919	0.620	0.778	0.891	0.893	0.915	0.609	0.654	0.897	0.902	0.918	0.624
	SP2	0.775					0.650					0.890				
	SP3	0.802					0.773					0.778				
	SP4	0.900					0.796					0.892				
	SP5	0.736					0.899					0.766				
	SP6	0.801					0.741					0.750				
	SP7	0.822					0.803					0.774

**Table 3:** Discriminant validity and HTMT

	Discriminant validity						HTMT					
		CEI	MC	EP	ENP	SP		CEI	MC	EP	ENP	SP
Pakistan	**CEI**	**0.782**					**CEI**					
	**MC**	0.496	**0.773**				**MC**	0.431				
	**ED**	0.454	0.589	**0.733**			**ED**	0.414	0.427			
	**END**	0.574	0.658	0.591	**0.841**		**END**	0.421	0.395	0.346		
	**SD**	0.525	0.554	0.544	0.583	**0.787**	**SD**	0.507	0.283	0.301	0.408	
Malaysia	**CEI**	**0.774**					**CEI**					
	**MC**	0.553	**0.748**				**MC**	0.505				
	**EP**	0.417	0.483	**0.813**			**EP**	0.407	0.403			
	**ENP**	0.576	0.564	0.461	**0.783**		**ENP**	0.529	0.539	0.443		
	**SP**	0.386	0.553	0.557	0.433	**0.780**	**SP**	0.344	0.474	0.355	0.245	
China	**CEI**	**0.768**					**CEI**					
	**MC**	0.475	**0.784**				**MC**	0.402				
	**EP**	0.413	0.456	**0.797**			**EP**	0.373	0.392			
	**ENP**	0.361	0.459	0.454	**0.786**		**ENP**	0.371	0.414	0.354		
	**SP**	0.588	0.442	0.371	0.457	**0.970**	**SP**	0.447	0.408	0.421	0.417	

Further, the procedure of variance inflation factor (VIF) was employed to assess multicollinearity among constructs (Rehman and Prokop 2023; Rehman et al. 2023b). However, the findings show that no issues of multicollinearity exist in the current data sets. Secondly, the study followed the step of structural model in PLS-SEM analysis procedure to test the defined hypothesis. The outcomes show that CEI and market competitiveness have positive significant relationship with the TBL efficiencies among the production SMEs as indicated in Table 4. Additionally, it was noted that CEI positively impacts market competitiveness in this study. The outcomes also indicate that market competitiveness mediates the association in CEI and the TBL efficiencies among the production SMEs in all the three countries (Table 5). Further, we found that the weight of Q-Square is non-zero that shows the existence of predictive relevance in the defined setting.

**Table 4:** Direct relationship (hypothesis testing)

Country	Relationship	Estimate	SM	SD	*T*-value	Decision	*R*-square	*F*-square	VIF	*Q*-square
Pakistan	CEI➔EP	0.214	0.212	0.053	4.084	Confirmed	0.246	0.056	1.323	0.194
	MC➔EP	0.482	0.486	0.049	9.842	Confirmed		0.284	1.327	
	CEI➔ENP	0.329	0.326	0.044	7.526	Confirmed	0.381	0.167	1.321	0.352
	MC➔ENP	0.495	0.497	0.043	11.600	Confirmed		0.379	1.320	
	CEI➔SP	0.332	0.33	0.056	5.926	Confirmed	0.514	0.136	1.326	0.227
	MC➔SP	0.389	0.391	0.055	7.104	Confirmed		0.187	1.332	
	CEI➔MC	0.496	0.498	0.044	11.354	Confirmed	0.390	0.327	1.324	0.139
Malaysia	CEI➔EP	0.266	0.263	0.038	6.999	Confirmed	0.306	0.145	1.420	0.154
	MC➔EP	0.636	0.639	0.031	20.451	Confirmed		0.528	1.440	
	CEI➔ENP	0.321	0.321	0.052	6.113	Confirmed	0.661	0.137	1.460	0.43
	MC➔ENP	0.463	0.465	0.046	9.995	Confirmed		0.286	1.450	
	CEI➔SP	0.325	0.325	0.052	6.239	Confirmed	0.481	0.146	1.480	0.282
	MC➔SP	0.473	0.475	0.048	9.919	Confirmed		0.311	1.410	
	CEI➔MC	0.553	0.556	0.041	13.490	Confirmed	0.499	0.440	1.000	0.29
China	CEI➔EP	0.512	0.515	0.047	10.974	Confirmed	0.226	0.345	1.272	0.126
	MC➔EP	0.213	0.215	0.050	4.301	Confirmed		0.106	1.290	
	CEI➔ENP	0.507	0.510	0.045	11.308	Confirmed	0.411	0.337	1.291	0.257
	MC➔ENP	0.218	0.218	0.049	4.473	Confirmed		0.062	1.282	
	CEI➔SP	0.488	0.490	0.048	10.159	Confirmed	0.409	0.298	1.274	0.255
	MC➔SP	0.211	0.210	0.049	4.286	Confirmed		0.055	1.269	
	CEI➔MC	0.475	0.482	0.044	10.794	Confirmed	0.38	0.291	1.297	0.232

**Table 5:** Indirect effects (hypothesis testing)

Country	Relationship	Estimate	SM	SD	*T*-value	CILL	CIUL	Decision
Pakistan	CEI➔MC➔EP	0.239	0.242	0.033	7.304	0.187	0.295	Confirmed
	CEI➔MC➔ENP	0.246	0.248	0.032	7.712	0.197	0.301	Confirmed
	CEI➔MC➔SP	0.193	0.195	0.034	5.743	0.145	0.248	Confirmed
Malaysia	CEI➔MC➔EP	0.351	0.355	0.029	12.19	0.300	0.408	Confirmed
	CEI➔MC➔ENP	0.256	0.259	0.029	8.694	0.201	0.316	Confirmed
	CEI➔MC➔SP	0.262	0.265	0.033	7.846	0.205	0.329	Confirmed
China	CEI➔MC➔EP	0.101	0.104	0.027	3.789	0.048	0.162	Confirmed
	CEI➔MC➔ENP	0.104	0.105	0.026	3.982	0.054	0.160	Confirmed
	CEI➔MC➔SP	0.106	0.101	0.026	3.895	0.054	0.154	Confirmed

**Fig. 2:**
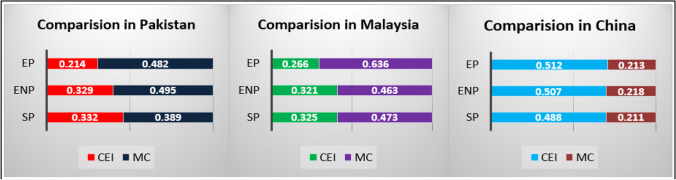
Comparison of CEI and MC

Surprisingly, the study noted that market competitiveness has greater effects on the TBL efficiencies among the production SMEs in Pakistan and Malaysia instead of CEI (Fig. 2). Interestingly, CEI has higher effects on the TBL efficiencies among the production SMEs in China as compared to market competitiveness. Due to the findings, we can say that market competitiveness has a much better role to enhance TBL efficiencies among the production SMEs in Malaysia and Pakistan as the firms in these countries may prefer the product and service innovation to deliver better services to gain the objectives of market competitiveness (Appendix 2). It is also possible that the production SMEs in Pakistan and Malaysia is preferring to establish the brand image and price minimization, as compared to the practices of CEI. It can also be said that the production SMEs in Pakistan and Malaysia are aggressively involved in differentiative marketing and designing competitive strategies as compared to CEI. Further, it can be said that the production SMEs in Pakistan and Malaysia are significantly focused on the internal operation system and manufacturing efficiencies to offer lower prices in market as compared to competitors.

Similarly, we observed that CEI has better role in China to achieve the TBL efficiencies among the production SMEs instead of market competitiveness (Appendix 2). Due to the reasons, we can assume that the Chinese production SMEs have adopted more innovative recyclable system for raw material and biodegradable inputs due to governmental policies and UN sustainability agenda. It can also be said that the production SMEs in China have already launched the innovative system to devalue the usage of non-recyclable raw material to implement the sustainability practices. It can also be inferred due to the outcomes that the production SMEs in China have initiated the environment friendly packaging system and using recyclable material as an input for their processes. Moreover, the findings have shown the goodness of model fit as in Table 6. The results have clarified that the values of SRMR and NFI exist in the acceptable range. As per the recommended threshold values, the SRMR should be in the range of “0 to 0.08,” and NFI should be in the range of “0 to 1.00” to achieve the objectives of goodness of model fit (Rizwanullah et al. 2021). Therefore, the objectives of goodness of model fit are achieved in this study.

**Table 6:** Comparison of model fit

	Pakistan		Malaysia		China	
	Saturated model	Estimated model	Saturated model	Estimated model	Saturated model	Estimated model
SRMR	0.076	0.105	0.092	0.135	0.076	0.16
d_ULS	4.098	7.746	5.906	12.826	4.045	18.035
d_G	1.813	2.042	2.536	3.229	3.489	4.602
Chi-square	2360.387	2520.693	3255.846	3771.083	4332.899	4975.211
NFI	0.752	0.735	0.693	0.644	0.619	0.562

## Discussion

This study aims to investigate the mediating role of market competitiveness between CEI and the TBL efficiencies among the production SMEs. The findings display that the practices of CEI and market competitiveness trigger TBL efficiencies among the production SMEs in emerging economies. The findings have verified that the CEI positively affect market competitiveness among the production SMEs. Further, the findings show that market competitiveness mediates between CEI and TBL efficiencies among the production SMEs. Hence, it has confirmed that the initiatives of CEI have the benefits to gain TBL efficiencies and market competitiveness among the production SMEs. The strategies of CEI can raise the service quality, build brand, improve profitability, and help to obtain the long-lasting objectives of TBL efficiencies among the production SMEs. The strategy of CEI can overcome the TBL deficiencies and tackle the issues of environmental degradation, pollution prevention, and energy savings, and is a helpful strategy to promote the trend of sustainable business practices.

The managerial concerns of the research were to investigate the nexus among defined variables and cover the gap of literature to enhance understanding in the TBL efficiencies among the production SMEs in the emerging economies. However, the outcomes of this study are parallel with Rehman et al. (2022) where the effects of CEI and business model innovation on the SDGs along with the mediating role of government incentives were evaluated. The results are inline due to the role of CEI in the SDGs among SMEs. The findings are also in line with Rehman et al. (2023b) where the association in CEI and digital sustainability was investigated. The results are parallel due to CEI and sustainability-oriented business practices. The outcomes of current study also confirmed the findings of Pieroni et al. (2019) who noted that circular economy positively influence on the economics, environmental, and social performance. Salvador et al. (2020) have noted that strategy of CE is a source of economic, environmental, and social values. Nussholz (2018) observed that the circular business model enhances the business, environmental, and social efficiencies. Stål and Corvellec (2018) found that the circular business model creates economic, environmental, and social values among firms. Likewise, the outcomes are also parallel to Ye et al. (2015) who examined the nexus between market competition and the economic, environmental, and social performance in the construction industry. Huang (2023) also found that market competition has a considerable role in the firm performance in China. Therefore, based on the current and previous findings, we can confirm that CEI and market competitiveness improve the TBL efficiencies among the production SMEs in emerging economies.

Moreover, the outcomes are in line with Anwar et al. (2018) who investigated the mediation of competitive advantage between business model innovation and firm performance. The results are also parallel to Rehman and Prokop et al. (2023) who observed that market competitiveness mediates between management practices and firm innovation. The outcomes are also in line with Mazzucchelli et al. (2022) who found that the brand reputation of firm mediates between the circular economy practices and firms’ financial performance. Therefore, based on the current and previous findings, we can confirm that market competitiveness mediates between CEI and the TBL efficiencies among the production SMEs in emerging economies. However, the results of the current study are exceptional due to investigating the role of CEI in the TBL efficiencies among the production SMEs in emerging economies in a comparative manner. The outcomes are also exclusive due to analyzing the mediating role of market competitiveness on the relationship between CEI and the TBL efficiencies among the production SMEs in emerging economies where the management of resources is usually a challenge for administration and sometimes needs to handle the unstructured issues in the day-by-day routines.

### Implications of the study

#### Implications for practices

The findings direct the practitioners to employ the strategies of CEI in the light of market competitiveness to achieve the TBL efficiencies among the production SMEs in emerging economies. The strategy of CEI in the light of market competitiveness can inspire the practitioners to follow the UN sustainability agenda along with the aim to achieve higher TBL efficiencies among the production SMEs and avoid from environmental penalties and legal issues. The regular practices of CEI can bring perfection in the skills set and improve confidence among employees that ultimately lead to gain market competitiveness and TBL efficiencies among the production SMEs. The perfection among skills set can lead the production SMEs toward the leadership position and build sustainability-oriented image among societies. The expertise in the practices of CEI can turn firms toward greater innovation, gain stakeholders trust, sustain market competitiveness, and achieve the TBL efficiencies. As the trust of stakeholders can increase the market shares, profit margin, and improve commitment toward TBL efficiencies among the production SMEs. The practices of CEI initiate the trend of recycling, reshaping, and reusing of material that can emerge the culture of circularity among the relevant industries and societies. The findings also guide the practitioners to understand the relative role of CEI in the factors of TBL efficiencies to provide better services among emerging economies. The outcomes can enable policy makers to wisely launch the initiatives of CEI to gain market competitiveness and obtain the sustainability-oriented objectives in business environment. Precisely, the findings of the study have inspired to integrate the strategies of CEI and market competitiveness to achieve the TBL efficiencies among production SMEs, specifically, in the emerging economies. Eventually, it also guides the practitioners to employ the wise resource management strategies in CEI to achieve market competitiveness, save the wastage of scare resources, and gain TBL efficiencies among the production SMEs in emerging economies. Therefore, the policymakers should focus on the wise resource management strategies in CEI to gain market competitiveness, reduce production cost, bring efficiency in supply chain system, and attain the TBL efficiencies among the production-based firms to ensure the UN sustainability-oriented objectives in the emerging economy. Particularly, it directs the policy makers in Pakistan, Malaysia, and China that the practices of CEI are important for both public and private sectors to reduce the greenhouse gases emissions, enhance market competitiveness, boost TBL efficiencies among the production SMEs, meet the corporate social responsibilities, and improve the sustainability-oriented image of their nations as per the UN agenda in global market.

#### Theoretical implications

The current research has contributed to the prior knowledge by integrating CEI and market competitiveness to achieve the TBL efficiencies among the production SMEs in emerging economies. It has also added in the earlier literature by validating the proposed research model based on the findings from emerging economies where the initiatives of CEI are rare in private sectors and SMEs face pressure from the external regulators due to greenhouse gases emissions. The findings also extend the RBV theory by validating the defined theoretical framework based on the collected data from the production SMEs among emerging economies. The validation of model improves the policy makers understanding to motivate SMEs in the initiatives of CEI to gain market competitiveness, reduce environmental deficiencies, avoid legal penalties, and attain TBL efficiencies. The findings contribute in RBV theory to utilize the internal capabilities to initiate the practices of CEI to achieve market competitiveness and TBL efficiencies.

Precisely, we evaluated the impacts of CEI and market competitiveness on TBL efficiencies among the production SMEs and proved based on the findings that CEI and market competitiveness trigger TBL efficiencies in the emerging economies. In our third contribution, we examine the effects of the CEI on the market competitiveness among the production SMEs in the emerging economies. The study proved that CEI triggers market competitiveness among the production SMEs in the emerging economies. In our fourth contribution, we evaluated the mediating role of market competitiveness between CEI and the TBL efficiencies among the production SMEs in emerging economies. The study confirmed that market competitiveness mediates between CEI and the TBL efficiencies among the production SMEs in emerging economies.

## Conclusion and future directions

The current research has investigated the role of CEI in TBL efficiencies along with the mediating role of market competitiveness among the production SMEs in emerging economies. A questionnaire-based survey technique was applied to collect data and analyzed through PLS-SEM. The findings discovered that CEI triggers market competitiveness and TBL efficiencies among SMEs and confirmed H1 and H2. Likewise, the findings indicated that market competitiveness triggers the TBL efficiencies among the production SMEs and confirmed H3. Similarly, the findings revealed that market competitiveness has a mediating role between CEI and TBL efficiencies among the production SMEs in emerging economies and confirmed H4 in this study. However, while generalizing the results, the practitioners should know about the scope of data collections. To further enrich the knowledge area, the future studies can investigate the mediating role of the management of resources, personality traits of the managers, business environment, and energy policies, business model innovation, management practices, R&D strategies, and digital capabilities. Future studies can also investigate the two-way relationship between the digital capabilities and market competitiveness along with environmental drivers. Future studies can investigate the role of CEI in corporate social responsibilities and firm internationalization to drive SMEs into global market. The future studies can investigate the role of control variable such as the age of firms, numbers of employees, and qualification of managers to further enrich the understanding of practitioners. To further brace the theoretical foundation, the future studies can apply the concept of contingency and stakeholders’ theories and specifically the application of ecological modernization theory to strengthen the sustainability-oriented image of firms among societies.

However, this study has the following limitations: first, it is only based on RBV while the addition of contingency, stakeholders’, and ecological modernization theories can show another impressive image of outcomes. This study has only assessed the mediating role of external factors while evaluating the mediating role of internal factors can alter the direction of policy makers.

## Figures and Tables

**Fig. 3:**
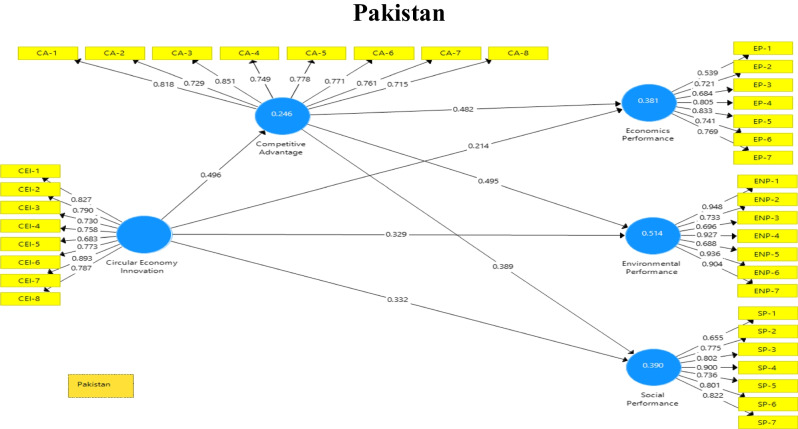
Pakistan

**Fig. 4:**
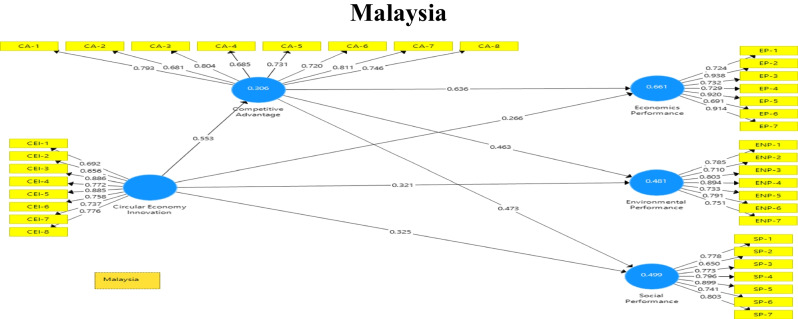
Malaysia

**Fig. 5:**
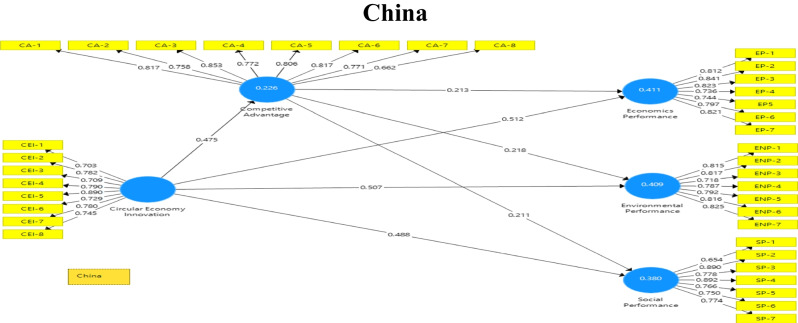
China

## Data Availability

Data will be provided on the request to the correspondence author.

## Appendix 1

See Tables 7, 8, and 9

**Table 7:** Cross-loading (Pakistan)

	Circular economy innovation	Market competitiveness	Economics performance	Environmental performance	Social performance
CEI-1	**0.827**	0.422	0.448	0.483	0.437
CEI-2	**0.790**	0.418	0.35	0.447	0.414
CEI-3	**0.730**	0.323	0.263	0.386	0.37
CEI-4	**0.758**	0.398	0.332	0.471	0.406
CEI-5	**0.683**	0.284	0.277	0.364	0.328
CEI-6	**0.773**	0.433	0.382	0.479	0.43
CEI-7	**0.893**	0.449	0.432	0.525	0.485
CEI-8	**0.787**	0.341	0.312	0.406	0.392
MC-1	0.345	**0.818**	0.493	0.493	0.402
MC-2	0.328	**0.729**	0.37	0.394	0.356
MC-3	0.381	**0.851**	0.519	0.467	0.408
MC-4	0.346	**0.749**	0.334	0.398	0.301
MC-5	0.356	**0.778**	0.367	0.457	0.344
MC-6	0.389	**0.771**	0.379	0.439	0.368
MC-7	0.472	**0.761**	0.553	0.583	0.483
MC-8	0.395	**0.715**	0.516	0.401	0.532
EP-1	0.209	0.243	**0.539**	0.244	0.249
EP-2	0.298	0.445	**0.721**	0.415	0.355
EP-3	0.306	0.322	**0.684**	0.359	0.352
EP-4	0.348	0.446	**0.805**	0.477	0.456
EP-5	0.478	0.536	**0.833**	0.578	0.539
EP-6	0.289	0.389	**0.741**	0.407	0.366
EP-7	0.335	0.536	**0.769**	0.461	0.405
ENP-1	0.507	0.587	0.54	**0.948**	0.767
ENP-2	0.529	0.531	0.466	**0.733**	0.666
ENP-3	0.376	0.471	0.441	**0.696**	0.453
ENP-4	0.488	0.576	0.509	**0.927**	0.528
ENP-5	0.468	0.369	0.459	**0.688**	0.315
ENP-6	0.497	0.559	0.533	**0.936**	0.474
ENP-7	0.482	0.548	0.521	**0.904**	0.506
SP-1	0.413	0.306	0.467	0.405	**0.655**
SP-2	0.398	0.41	0.404	0.533	**0.775**
SP-3	0.397	0.424	0.434	0.439	**0.802**
SP-4	0.438	0.452	0.45	0.471	**0.900**
SP-5	0.363	0.359	0.374	0.363	**0.736**
SP-6	0.357	0.43	0.434	0.422	**0.801**
SP-7	0.372	0.422	0.397	0.442	**0.822**

**Table 8:** Cross-loading (Malaysia)

	Circular economy innovation	Market competitiveness	Economics performance	Environmental performance	Social performance
CEI-1	**0.692**	0.404	0.398	0.33	0.377
CEI-2	**0.656**	0.409	0.449	0.422	0.446
CEI-3	**0.886**	0.476	0.526	0.496	0.501
CEI-4	**0.772**	0.391	0.471	0.434	0.425
CEI-5	**0.885**	0.482	0.532	0.486	0.486
CEI-6	**0.758**	0.388	0.446	0.43	0.408
CEI-7	**0.737**	0.399	0.427	0.42	0.421
CEI-8	**0.776**	0.457	0.443	0.318	0.337
MC-1	0.373	**0.793**	0.493	0.373	0.404
MC-2	0.328	**0.681**	0.398	0.323	0.365
MC-3	0.394	**0.804**	0.471	0.389	0.442
MC-4	0.305	**0.685**	0.392	0.275	0.309
MC-5	0.319	**0.731**	0.447	0.319	0.353
MC-6	0.423	**0.720**	0.465	0.327	0.347
MC-7	0.496	**0.811**	0.385	0.398	0.477
MC-8	0.417	**0.746**	0.361	0.412	0.319
EP-1	0.412	0.414	**0.724**	0.573	0.476
EP-2	0.566	0.427	**0.938**	0.591	0.464
EP-3	0.355	0.493	**0.732**	0.475	0.454
EP-4	0.414	0.449	**0.729**	0.318	0.548
EP-5	0.531	0.408	**0.92**	0.469	0.472
EP-6	0.466	0.309	**0.691**	0.407	0.412
EP-7	0.547	0.412	**0.914**	0.477	0.342
ENP-1	0.453	0.456	0.456	**0.785**	0.463
ENP-2	0.375	0.383	0.549	**0.71**	0.472
ENP-3	0.466	0.503	0.436	**0.803**	0.338
ENP-4	0.489	0.533	0.438	**0.894**	0.265
ENP-5	0.413	0.458	0.313	**0.733**	0.476
ENP-6	0.539	0.477	0.475	**0.791**	0.321
ENP-7	0.376	0.412	0.254	**0.751**	0.468
SP-1	0.408	0.491	0.508	0.496	**0.778**
SP-2	0.551	0.566	0.497	0.456	**0.65**
SP-3	0.441	0.494	0.459	0.407	**0.773**
SP-4	0.449	0.497	0.477	0.347	**0.796**
SP-5	0.499	0.543	0.375	0.481	**0.899**
SP-6	0.401	0.432	0.591	0.433	**0.741**
SP-7	0.407	0.501	0.551	0.362	**0.803**

**Table 9:** Cross-loading (China)

	Circular economy innovation	Market competitiveness	Economics performance	Environmental performance	Social performance
CEI-1	**0.703**	0.377	0.471	0.478	0.424
CEI-2	**0.782**	0.336	0.461	0.457	0.454
CEI-3	**0.709**	0.267	0.44	0.422	0.38
CEI-4	**0.790**	0.385	0.451	0.45	0.473
CEI-5	**0.890**	0.400	0.53	0.524	0.494
CEI-6	**0.729**	0.324	0.42	0.424	0.419
CEI-7	**0.780**	0.469	0.567	0.555	0.545
CEI-8	**0.745**	0.316	0.388	0.403	0.386
MC-1	0.381	**0.817**	0.378	0.401	0.37
MC-2	0.333	**0.758**	0.345	0.342	0.321
MC-3	0.397	**0.853**	0.366	0.385	0.385
MC-4	0.275	**0.772**	0.303	0.293	0.301
MC-5	0.327	**0.806**	0.329	0.324	0.307
MC-6	0.337	**0.817**	0.347	0.336	0.332
MC-7	0.321	**0.771**	0.296	0.295	0.283
MC-8	0.415	**0.662**	0.435	0.431	0.413
EP-1	0.466	0.307	**0.812**	0.431	0.305
EP-2	0.492	0.356	**0.841**	0.483	0.304
EP-3	0.533	0.367	**0.823**	0.266	0.464
EP-4	0.465	0.429	**0.736**	0.586	0.357
EP-5	0.483	0.359	**0.744**	0.497	0.442
EP-6	0.493	0.381	**0.821**	0.445	0.478
EP-7	0.482	0.340	**0.797**	0.608	0.401
ENP-1	0.473	0.391	0.427	**0.815**	0.575
ENP-2	0.489	0.361	0.441	**0.817**	0.451
ENP-3	0.49	0.326	0.491	**0.718**	0.593
ENP-4	0.501	0.376	0.483	**0.787**	0.403
ENP-5	0.503	0.373	0.376	**0.792**	0.443
ENP-6	0.467	0.368	0.319	**0.816**	0.668
ENP-7	0.474	0.358	0.424	**0.825**	0.539
SP-1	0.422	0.355	0.357	0.461	**0.654**
SP-2	0.499	0.383	0.449	0.449	**0.890**
SP-3	0.437	0.302	0.465	0.465	**0.778**
SP-4	0.489	0.398	0.376	0.443	**0.892**
SP-5	0.423	0.294	0.428	0.327	**0.766**
SP-6	0.427	0.337	0.402	0.379	**0.750**
SP-7	0.232	0.361	0.411	0.389	**0.774**

## Appendix 2

See Figs. 3, 4, and 5
